# Feline Rotavirus A as a Source of Spillover Infections to Humans: An In-Depth Analysis of Molecular Epidemiological Evidence

**DOI:** 10.3390/v18020207

**Published:** 2026-02-05

**Authors:** Osamu Nakagomi, Toyoko Nakagomi

**Affiliations:** Department of Hygiene and Molecular Epidemiology, Graduate School of Biomedical Sciences, Nagasaki University, Nagasaki 852-8523, Japan

**Keywords:** rotavirus A, feline rotavirus, canine rotavirus, interspecies transmission, genogroup, genotype constellation, reassortment, fitness landscape, whole-genome sequencing

## Abstract

Rotavirus A (RVA) is a leading cause of severe diarrhoea in children, and interspecies transmission significantly drives the genomic diversity of human RVAs. Cats represent a key host species, requiring in-depth analysis regarding RVA transmission to humans. This review evaluated the literature on the complex genotype constellations of feline RVAs in relation to relevant canine and human RVAs to define the role of feline RVAs in the evolutionary history of human strains. The review traces the methodological shift from genogrouping by RNA-RNA hybridisation to the current genotype constellation system enabled by whole-genome sequencing. While early methods identified a shared genomic closeness between human AU-1 and feline FRV-1, whole-genome sequencing indicated that several human RVA strains, including AU-1, HCR3A, and Ro1845, likely resulted from direct transmission of feline/canine strains, due to shared genotype constellations and high sequence identity with animal strains like feline FRV-1, Cat97 and canine CU-1. Evidence of reassortment—such as the emergence of G1P[9] and G9P[9] strains after the feline-derived G3P[9] crossed into the human population—suggests these feline-like strains have successfully overcome the host-species barrier and are capable of onward human-to-human transmission, not just dead-end spillover events. However, definitive confirmation of sustained transmission or contemporary spillover requires stringent phylogenetic criteria: multiple human strains with >99% identical sequences in a monophyletic lineage for sustained transmission, or an identical human–feline pair across all genome segments for contemporary spillover. Confirming the status of the AU-1-like constellation as a third, low-frequency human RVA type requires future studies applying these strict criteria.

## 1. Introduction

### 1.1. Taxonomic Status of Rotavirus A

Genus *Rotavirus* within the *Sedoreoviridae* family contains nine species, of which *Rotavirus alphagastroenteritidis*, more commonly called rotavirus A (RVA), was responsible for the vast majority of the severe diarrhoea in infants and young children prior to the introduction of rotavirus vaccines [1,2,3].

### 1.2. Virological Features of Rotavirus A

RVA measures 75 nm in diameter and exhibits a wheel-like structure with icosahedral symmetry when viewed under negative-stain electron microscopy [3]. This structure consists of a three-layered capsid: the outermost layer is a smooth, rounded surface composed of VP7 proteins and features VP4 spike proteins that protrude from it. Both VP7 and VP4 carry neutralisation antigens which define the G and P types, respectively. The middle-layer capsid is composed of most abundant VP6 proteins. The innermost layer is formed by VP2 proteins. This innermost layer constitutes the core, which contains the VP1/VP3 replication enzyme complex and the genomic RNA. The RVA genome is made up of 11 segments of double-stranded RNA, each of which codes for six viral structural proteins (VP1–VP4, VP6 and VP7) and six nonstructural proteins (NSP1–NSP5/6). The rotavirus gene is monocistronic except for gene 11 which codes for two in-frame nonstructural proteins, NSP5 and NSP6 [3].

### 1.3. Genetic Diversity of Rotavirus A Genome: Genotype and Genotype Constellation

Each gene of the rotavirus A genome exhibits a certain degree of nucleotide sequence diversity, which has become apparent as data have accumulated through the widespread use of whole-genome sequencing [4]. Accordingly, Matthijnssens et al. [4] defined genotypes based on nucleotide sequence identity cutoff values: VP1 (83%), VP2 (84%), VP3 (81%), VP4 (80%), VP6 (79%), VP7 (85%), NSP1 (85%), NSP2 (85%), NSP3 (80%), NSP4 (85%), and NSP5/6 (91%) (Table 1).

Furthermore, human rotavirus strains are grouped into three distinct sets of genotype constellations: two major (Wa-like and DS-1-like) and one minor (AU-1-like) [4,5]. More specifically, Wa-like, DS-1-like and AU-1-like genotype constellations represent G1/G3/G4/G9/G12-P[8]-I1-R1-C1-M1-A1-N1-T1-E1-H1, G2-P[4]-I2-R2-C2-M2-A2-N2-T2-E2-H2, and G3-P[9]-I3-R3-C3-M3-A3-N3-T3-E3-H3, respectively (Table 2) [6].

### 1.4. Diverse Host Range and Interspecies Transmission

RVA has a wide host range, as indicated by its isolation from the newborn and young of many animal species [7]. However, RVA strains infect particular host animal species preferentially to others, and consequently, they are called the homologous strains. Heterologous RVA infections may occur in both natural and experimental conditions [8]. It is now generally accepted that interspecies transmission contributes to the rotavirus genomic diversity upon which adaptive pressures exert to select a better-fit progeny subpopulation for survival.

Among the domestic and wild animal sources of RVA strains capable of cross-species transmission, cats occupy a unique research niche. First, initial molecular evidence of interspecies transmission was established by demonstrating that the unusual human RVA strain, AU-1, was indistinguishable from the feline strain FRV-1 [9]. Second, the subsequent isolation of AU-1-like strains [10] supported the proposal of a third human RVA group, distinct from the more common Wa-like and DS-1-like genotypes [11]. This finding implies that RVA strains of feline origin can jump to humans and continue to circulate at low frequencies within the human population. Third, despite the limited number of reported strains, research over the last four decades has identified a variety of genotype constellations [12,13,14,15,16,17,18]. Because this inquiry spanned several decades, the molecular epidemiological evidence is naturally subject to the methodological limitations of each era. Consequently, an in-depth analysis is required to distinguish between isolated spillover events and sustained human-to-human transmission following an interspecies jump.

A clarification regarding the nomenclature of “-like” strains is necessary for this review. By convention, we define human, feline, and canine rotaviruses as those detected in humans, cats, and dogs, respectively, regardless of the host species suggested by their genomic characteristics. In the literature, a rotavirus strain with genomic sequences similar to feline RVA is referred to as a “feline-like human”; we follow this convention where appropriate. In other contexts, we use the suffix “-like” to indicate similarity to a reference strain’s genomic composition (typically possessing an identical genotype constellation), such as AU-1-like strains. Consequently, the interpretation of “-like” is context-dependent.

### 1.5. The Aim of the Review

The specific aims of this review are threefold: firstly, to clarify the evolution of operational tools for whole-genome analysis of RVA, specifically the transition from genogrouping to genotype constellation analysis enabled by the shift from RNA-RNA hybridization to whole-genome sequencing; secondly, to re-evaluate how interspecies transmission has been operationally defined and applied within key exemplary cases; and thirdly, to systematically review and organize the literature regarding the complex genotype constellations of feline and canine RVA strains, adding feline-like or feline/canine-like human strains, and provide an updated reference table of these constellations for researchers in the field.

By integrating information thus far available, our ultimate goal is to reveal the critical knowledge gaps and establish a framework for defining the role of feline rotaviruses in the evolutionary history of human rotaviruses.

## 2. The Operational Role of the Concept of Genogroup in Addressing Interspecies Transmission and Its Methodological Limitations

When addressing interspecies transmission in the context of human rotavirus molecular epidemiology, the primary challenge lies in identifying a heterologous strain among a vast majority of homologous clinical samples. To address this, the concept of the genogroup, as determined by RNA-RNA hybridization, was introduced [11]. Broadly, RVA strains originating from a specific host species typically belong to a single genogroup or a limited number of genogroups; conversely, strains from different animal hosts rarely share the same genogroup [19,20,21,22,23]. Consequently, cases where different animal rotaviruses share a genogroup are interpreted as molecular evidence of interspecies transmission.

**Figure 1:**
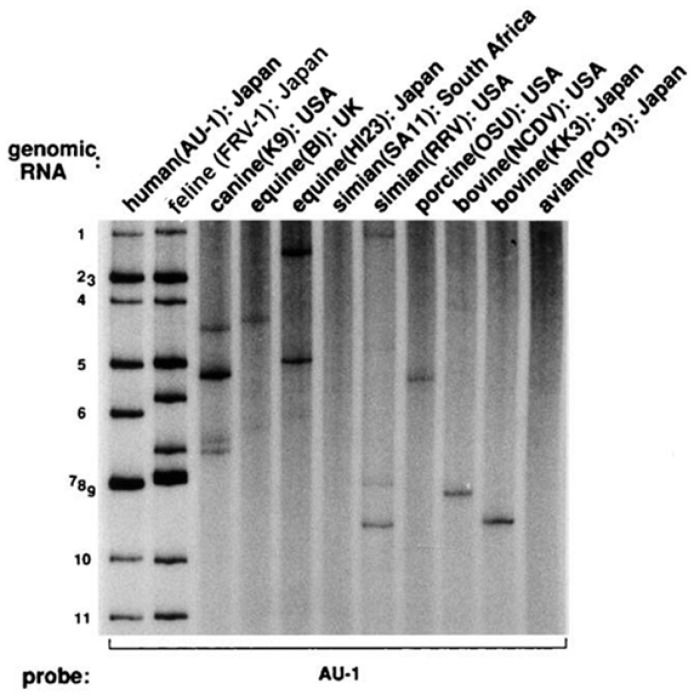
Genogrouping by RNA–RNA hybridisation of the AU-1 strain in comparison with genomic RNAs from various animal rotavirus strains. Only the genomic RNA from a feline rotavirus from Japan (the FRV-1 strain) shows a hybridisation pattern that is indistinguishable from the homologous reaction pattern generated by the AU-1 probe and the AU-1 genome. Conversely, the probe failed to significantly hybridise with the genomes of other mammalian and avian rotaviruses derived from six different host animal species. This observation formed the basis of a paradigm that the sharing of a genogroup by rotaviruses of different host species origin is taken as molecular evidence for rotavirus transmission across the host species barrier. Approximate positions of the genome segments of the AU-1 strain are indicated to the left of the panel. Reproduced from ref. [23] with permission from the publisher.

For example, Figure 1 demonstrates that a probe derived from the human AU-1 strain hybridized with the genomic RNA of the feline FRV-1 strain in a manner indistinguishable from its hybridization with AU-1 itself. This finding strongly suggests that AU-1 originated from a feline RVA [8]. While the genogroup concept was instrumental in defining the three human RVA genogroups (Wa, DS-1, and AU-1) [11], the method has significant limitations: it cannot identify homology at the level of individual genome segments, nor can it distinguish between contemporary spillover events and the sharing of a distant common ancestor.

## 3. The Shift to Whole-Genome Sequencing and Defining Genotype Constellation as a New Operational Tool

### 3.1. From Genogroup to Genotype Constellation

The limitations of genogrouping were eventually overcome by whole-genome sequencing which was pioneered by Matthijnssens et al. [24]. This technology enables high-resolution phylogenetic analysis of all eleven genome segments directly from minimal faecal samples. Consequently, the entire genome can be summarized as a genotype constellation, as previously described (Section 1.3).

### 3.2. Operational Criteria for Spillover

Earlier, Matthijnssens et al. [24] sequenced the whole genome of two strains: a Belgian rotavirus strain B4106, isolated from a child with gastroenteritis, and a European G3P[14] rabbit rotavirus strain 30/96. They found the overall nucleotide sequence identity between the two strains was 93.4%, ranging from a low of 88.2% in the VP2 gene to a high of 96.9% in the VP6 gene. Furthermore, phylogenetic analyses showed all 11 genome segments of strain B4106 clustered with the rabbit strains. The two strains shared the same genotype constellation of G3-P[14]-I2-R2-C2-M3-A9-N2-T6-E5-H3. This confirmed that the human strain B4106 possessed an entirely rabbit genome complement and was capable of infecting and causing disease in a human child. Thus, the B4106 strain represents a direct interspecies transmission (spillover) event of a fully rabbit-adapted virus, rather than a reassortant strain.

The criteria that Matthijnssens et al. [24] used in this study for determining a given strain to be directly derived from a heterologous host (interspecies transmission as whole virions) can be summarised twofold: (i) high nucleotide sequence identities across the 11 gene segments between the test strain and putative original host-derived RVA strains (no particular cutoff value mentioned), and (ii) the clustering of its sequence with sequences of putative original host-derived RVA strains in phylogenetic analysis. In essence, the criteria are not significantly more granular than genogrouping by RNA-RNA hybridisation—a method that theoretically permitted up to 18% nucleotide mismatches to form stable hybrid bands [25]. While these criteria provide far higher resolution regarding gene segment identification, they consequently fail to fully leverage the quantitative metrics of whole-genome sequencing. In a later section (Section 7), we will revisit and propose the criteria that should be employed.

### 3.3. How the Operational Criteria Were Used: Case Examples

Let us now examine how these two criteria were employed by other investigators to designate a strain as having originated from a contemporary spillover event of feline or feline/canine rotaviruses, using whole-genome sequencing data.

#### 3.3.1. Human Ro1845 and HCR3A Strains

The first case involves two human RVA strains Ro1845 and HCR3A, isolated in Israel in 1985 and in Philadelphia, U.S.A., in 1984, respectively, that resulted from direct virion transmission of canine/feline RVA strains to humans [26,27]. Tsugawa and Hoshino sequenced the whole genomes of three canine strains, CU-1, K9 and A79-10, and one feline strain, Cat97, in addition to Ro1845 and HCR3A. All of them had the same G3-P[3]-I3-R3-C2-M3-A9-N2-T3-E3-H6 constellation, showed 94–99% nucleotide sequence identities, and clustered together in the phylogenetic trees of all genes. With these findings, which clearly corresponded to the two criteria used by Matthijnssesns et al. [24], Tsugawa and Hoshino concluded that Ro1845 and HCR3A were of canine/feline origin [27]. Following this conclusion, they further stated that CU-1, K9, A79-10, and Cat97 were recovered from a homologous host (dogs or cats). This notion contained a deeper interpretation that dogs and cats are considered equivalent homologous host species from the perspective of RVA strains possessing this specific genotype constellation. To discuss the implication of this notion, it is necessary to employ the concept of fitness landscape, as we detail in a later section (Section 5).

#### 3.3.2. Human AU-1 and Feline FRV-1 Strains

The second case involves the human AU-1 and feline FRV-1 strains, which offered the first compelling evidence that an animal rotavirus could successfully infect humans and potentially cause disease under natural conditions [9,22]. The sharing of the genogroup between AU-1 and FRV-1 was confirmed by whole-genome sequencing to show an identical genotype constellation of G3-P[9]-I3-R3-C3-M3-A3-N3-T3-E3-H3 between two strains with high nucleotide sequence identities ranging between 88.9% and 99.5% [28,29]. Thies identities were considered sufficiently high for AU-1 to be regarded as an example of direct transmission of a feline rotavirus to humans, meeting the high sequence identity criterion established, albeit implicitly, by Matthijnssens et al. [24].

While both strains belong to the same genotype for all genes, a more granular analysis by Gauchan et al. [28] revealed some divergence between FRV-1 and AU-1 in three genes. Specifically, the VP7, VP6 and VP2 genes showed a divergence of 5.8%, 7.1% and 11.1%, respectively. Despite belonging to different lineages within their respective genotypes, Gauchan, et al. [28] elaborated on why their conclusion of direct transmission remains appropriate, resting on the precedence of other feline-like strains in the literature. The sequence identity observed between FRV-1 and AU-1 (divergence of 0.6–11.1%) is comparable to the divergence seen between the feline strain BA222 [12] and other human rotavirus strains such as Italian PAH136/96 [30,31], Tunisian 17237 [32], and Japanese KF17 [33] that are already considered results of direct transmission from a BA222-like feline rotavirus. Here, the BA222-like G3/G6P[9] feline rotavirus strain represents the proposed third genotype constellation of feline rotaviruses [13], as discussed in the next section (Section 4). For these precedents, nucleotide sequence divergence ranged from 0.3% to 15.4%. In addition, Gauchan et al. [28] pointed out that the full-genome sequencing of more FRV-1-like feline rotavirus strains, when available, will reveal a natural degree of sequence diversity, resulting in lineages different from the original FRV-1 strain, thereby justifying the observed difference.

#### 3.3.3. Human PA260-97 and Canine 135 Strains

The third case involves the pair of human G3P[3] strain PA260-97 detected in Italy and canine G3P[3] strain 135 detected in Hungary, both of which possessed the G3-P[3]-I3-R3-C3-M3-A15-N2-T3-E3-H3 constellation and nucleotide sequence identities ranging from 98.3% to 99.2% [13,34]. Together with the sharing of a rare NSP1 genotype, A15, the study provided strong evidence of direct interspecies transmission of a canine rotavirus to a human.

In conclusion, the advent of metagenomics, whole-genome sequencing, and advanced phylogenetic analyses necessitates questioning whether criteria not significantly advanced beyond the older model of genogrouping by RNA-RNA hybridisation are still adequate.

## 4. Complex Genomic Diversity of Feline Rotaviruses

### 4.1. Diverse Feline Genotype Constellations

As of this writing, feline RVA strains comprise nine distinct genotype constellations, which can be categorized into three or four major groups and several reassortant variants (Table 3). The AU-1/FRV-1-like (G3-P[9]-I3-R3-C3-M3-A3-N3-T3-E3-H3) and Cat97-like (G3-P[3]-I3-R3-C2-M3-A9-N2-T3-E3-H6) constellations are the most clearly defined. However, whether the BA222-like and Meesek/Mie-like constellations (G3/G6-P[9]-I2-R2-C2-M2-A3-N2-T3-E2-H3) represent distinct groups or a single cluster remains a subject of debate. A notable exception is the FRV537 strain, which resulted from a direct spillover of a G6P[5] bovine RVA into a stray cat [18].

### 4.2. Interpretation of Cat2 as Reassortant

Interpreting this complex diversity requires acknowledging the significant roles of reassortment and interspecies transmission. For instance, Cat2, an Australian prototype strain isolated alongside Cat97, appears to be a natural reassortant primarily between Cat97-like and BA222-like ancestors [13]. The generation of the Cat2 genotype constellation was discussed extensively by Gauchan et al. [28] once the full FRV-1 sequence became available. They concluded that Cat2 was not a simple reassortant between just two strains (e.g., FRV-1 and FRV64, or Cat97 and BA222). Instead, they provided evidence for multiple reassortment events having occurred throughout the course of Cat2’s evolution. However, because the T6 genotype is absent in all feline RVAs except for the bovine-origin FRV537, the emergence of strains like Cat2 cannot be explained by simple reassortment among known feline RVAs alone. Significantly, Gauchan et al. [28] suggested that Cat2 is unlikely to be the only feline rotavirus strain that has undergone reassortment.

More importantly, however, the observation that FRV-1 and BA222 have closely related sequences at the lineage level in the VP7 (G3), VP4 (P[9]), NSP1 (A3), and NSP3 (T3) genes suggests that these genome segments were possessed by the same ancestral strain in the recent past [28].

**Table 3:** Genotype constellations of feline and canine RVA strains together with those of relevant human RVA strains.

Host		Strain	Country	Year	VP7	VP4	VP6	VP1	VP2	VP3	NSP1	NSP2	NSP3	NSP4	NSP5	Ref.
Cats	FRV-1-like	FRV-1	JPN	1985	G3	P[9]	I3	R3	C3	M3	A3	N3	T3	E3	H3	[28]
		FRV317	JPN	1994	G3	P[9]	I3	R3	C3	M3	A3	N3	T3	E3	H3	[16]
		FRV384	JPN	1994	G3	P[9]	I3	R3	C3	M3	A3	N3	T3	E3	H3	[16]
	Unique	Cat2	AUS	1984	G3	P[9]	I3	R3	C2	M3	A3	N1	T6	E3	H6	[27]
	BA22/Mie-like	BA222	ITA	2005	G3	P[9]	I2	R2	C2	M2	A3	N1	T3	E2	H3	[12]
		Meesuk/2021	THA	2021	G3	P[9]	I2	R2	C2	M2	A3	N2	T3	E3	H3	[14]
		Mie20120017f	JPN	2012	G6	P[9]	I2	R2	C2	M2	A3	N2	T3	E3	H3	[17]
		Mie20120022f	JPN	2012	G6	P[9]	I2	R2	C2	M2	A3	N2	T3	E3	H3	[17]
	Cat97-like	Cat97	AUS	1984	G3	P[3]	I3	R3	C2	M3	A9	N2	T3	E3	H6	[27]
		FRV64	JPN	1990	G3	P[3]	I3	R3	C2	M3	A9	N2	T3	E3	H6	[28]
		FRV72	JPN	1990	G3	P[3]	I3	R3	C2	M3	A9	N2	T3	E3	H6	[16]
		FRV73	JPN	1990	G3	P[3]	I3	R3	C2	M3	A9	N2	T3	E3	H6	[16]
		FRV303	JPN	1994	G3	P[3]	I3	R3	C2	M3	A9	N2	T3	E3	H6	[16]
	Unique	FRV348	JPN	1994	G3	P[3]	I3	R3	C3	M3	A15	N3	T3	E3	H6	[16]
	Unique	CU25045	THA	2020	G3	P[3]	I8	R3	C3	M3	A9	N3	T3	E3	H6	[35]
	Bovine-like	FRV537	JPN	2004	G6	P[5]	I2	R2	C2	M2	A13	N2	T6	E2	H6	[18]
Dogs	Cat97/CU-1-like	CU-1	USA	1982	G3	P[3]	I3	R3	C2	M3	A9	N2	T3	E3	H6	[27]
		K9	USA	1979	G3	P[3]	I3	R3	C2	M3	A9	N2	T3	E3	H6	[27]
		A79-10	USA	1979	G3	P[3]	I3	R3	C2	M3	A9	N2	T3	E3	H6	[27]
		RV198-95	ITA	1995	G3	P[3]	I3	R3	C2	M3	A9	N2	T3	E3	H6	[13]
		RV52-96	ITA	1996	G3	P[3]	I3	R3	C2	M3	A9	N2	T3	E3	H6	[13]
		135	HUN	2012	G3	P[3]	I3	R3	C3	M3	A15	N2	T3	E3	H6	[34]
		RS-15	JPN	1983	G3	P[3]	I3	R3	C2	M3	A9	N3	T3	E3	H6	[35]
		CU25012	THA	2020	G3	P[3]	I3	R3	C3	M3	A9	N2	T3	E3	H6	[35]
Humans	Feline-like	AU-1	JPN	1982	G3	P[9]	I3	R3	C3	M3	A3	N3	T3	E3	H3	[29]
		AU1115	JPN	1986	G3	P[9]	I3	R3	C3	M3	A3	N3	T3	E3	H3	[15]
		R47	BRA	1997	G3	P[9]	I3	R3	C3	M3	A3	N3	T3	E3	H3	[36]
		R55	BRA	1997	G3	P[9]	I3	R3	C3	M3	A3	N3	T3	E3	H3	[36]
		R57	BRA	1997	G3	P[9]	I3	R3	C3	M3	A3	N3	T3	E3	H3	[36]
		R142	BRA	1999	G3	P[9]	I3	R3	C3	M3	A3	N3	T3	E3	H3	[36]
		R138	BRA	1998	G9	P[9]	I3	R3	C3	M3	A3	N1	T1/T3	E3	H3	[36]
		R135	BRA	1998	G9	P[9]	I1	R1	C1	M1	A1	N1	T2	E1	H1	[36]
		R70	BRA	1997	G1	P[9]	I1	R1	C1	M3	A1	N1	T2	E1	H1	[36]
		R49	BRA	1997	G1	P[9]	I1	R1	C1	M2	A1	N2	T2	E1	H1	[36]
		WZ606	CHN	2013	G3	P[9]	I3	R3	C3	M3	A3	N3	T3	E3	H3	[37]
		K8	JPN	1977	G1	P[9]	I1	R3	C3	M3	A1	N1	T3	E3	H3	[38]
		PAI58/96	ITA	1996	G3	P[9]	I2	R2	C2	M2	A3	N2	T6	E2	H3	[31]
		PAH136/96	ITA	1996	G3	P[9]	I2	R2	C2	M2	A3	N1	T6	E2	H3	[31]
		GER29-14	DEU	2014	G6	P[9]	I2	R2	C2	M2	A3	N2	T3	E2	H3	[39]
		Se584	USA	1998	G6	P[9]	I2	R2	C2	M2	A3	N2	T1	E2	H3	[40]
		CC425	USA	1998	G3	P[9]	I2	R2	C2	M2	A3	N2	T1	E2	H3	[41]
		CU365	THA	2008	G3	P[9]	I3	R3	C3	M3	A3	N3	T3	E3	H6	[42]
		2020999	CHN	2020	G3	P[9]	I2	R2	C2	M2	A3	N2	T3	E3	H3	[43]
		23582009	CHN	2020	G3	P[9]	I2	R2	C2	M2	A3	N2	T3	E3	H3	[43]
		N566	Leba	2011	G3	P[9]	I2	R2	C2	M2	A3	N2	T1	E2	H2	[44]
		N235	Leba	2013	G3	P[9]	I2	R2	C2	M2	A3	N1	T2	E2	H1	[44]
		12US1134	USA	2012	G3	P[9]	I2	R2	C2	M2	A3	N2	T1	E2	H3	[45]
		0537	USA	2002	G3	P[9]	I2	R2	C2	M2	A3	N2	T1	E2	H3	[46]
		CAU12-2-51	KOR	2012	G3	P[9]	I2	R2	C2	M2	A3	N2	T3	E3	H3	[47]
		CAU14-1-262	KOR	2014	G3	P[9]	I3	R3	C3	M3	A3	N3	T1	E3	H6	[48]
		R11-035	JPN	2015	G3	P[9]	I3	R3	C3	M3	A3	N3	T1	E3	H6	[15]
		L621	CHN	2006	G3	P[9]	I3	R3	C3	M3	A3	N3	T3	E3	H6	[49]
		E2451	CHN	2011	G3	P[9]	I3	R3	C3	M3	A3	N3	T3	E3	H6	[49]
		KF17	JPN	2010	G6	P[9]	I2	R2	C2	M2	A3	N2	T3	E3	H3	[33]
		17237	TUN	2008	G6	P[9]	I2	R2	C2	M2	A3	N1	T6	E2	H3	[32]
		T152	THA	1998	G12	P[9]	I3	R3	C3	M3	A12	N3	T3	E3	H3	[50]
	Unique	ME848/12	ITA	2012	G12	P[9]	I17	R12	C12	M11	A12	N12	T7	E6	H2	[51]
	Feline/canine-like	PA260-97	ITA	1997	G3	P[3]	I3	R3	C3	M3	A15	N2	T3	E3	H6	[13]
		6212	USA	2003	G3	P[3]	I3	R1	C2	M3	A9	N2	T3	E3	H6	[46]
		6235	USA	2003	G3	P[3]	I2	R1	C2	M3	A9	N2	T3	E3	H6	[46]
		HCR3A	USA	1984	G3	P[3]	I3	R3	C2	M3	A9	N2	T3	E3	H6	[27]
		Ro1845	ISR	1985	G3	P[3]	I3	R3	C2	M3	A9	N2	T3	E3	H6	[27]
		12638	JPN	2014	G3	P[3]	I3	R3	C3	M3	A9	N2	T3	E3	H6	[52]
		WZ101	CHN	2013	G3	P[3]	I3	R3	C3	M3	A9	N3	T3	E3	H6	[37]

### 4.3. Interpretation of G6P[9] Feline RVA Strains

The sporadic detection of human G6P[9] strains—such as KF17 in Japan [33], 17237 in Tunisia [32], and GER29-14 in Germany [39]—with the genotype constellation G6-P[9]-I2-R2-C2-M2-A3-N2-T3-E2-H3 was initially attributed to reassortment between bovine and AU-1-like feline RVAs. This interpretation stemmed from the long-standing observation that feline RVA VP7 genotypes were almost exclusively G3.

However, this perception shifted following a nationwide molecular epidemiological survey in the United Kingdom by German et al. [53], which revealed that the majority of feline RVAs carry the G6P[9] genotype. This shift was further reinforced by the characterization of two Japanese feline G6P[9] strains, Mie20120017f and Mie20120022f. These strains possess a genotype constellation identical to the human KF17 strain (G6-P[9]-I2-R2-C2-M2-A3-N2-T3-E2-H3), strongly suggesting that human G6P[9] RVAs result from direct spillover events from feline populations rather than complex inter-species reassortment.

## 5. Interspecies Transmission and Host Adaptation

### 5.1. Spillover, Adaptation, and the Fitness Landscape

Current consensus suggests that the isolation of a human RVA strain indistinguishable from those found in an animal host represents a case of direct whole virion transmission (a spillover event) rather than a strain already circulating endemically within the human population. While our understanding of these events is limited by narrow sampling windows of global viral diversity, it raises a critical question: is it feasible for a single strain to achieve dual host adaptation? Specifically, can an RVA strain evolve to replicate equally efficiently in two different host species, thereby simultaneously maintaining sustained inter-individual transmission chains within both host populations? This question is an issue that impacts the interpretation of the AU-1-like strains as a potential third human rotavirus genotype constellation.

### 5.2. Evidence for Dual Host Adaptation of Cat97-like Strains

#### 5.2.1. The Concept of Fitness Landscape

**Figure 2:**
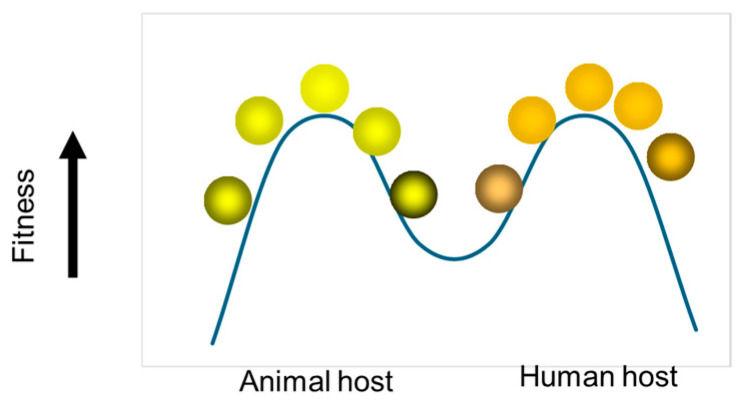
The concept of the fitness landscape. The concept of the fitness landscape is crucial for understanding how rotavirus strains adapt, persist, or go extinct after an interspecies transmission event. The fitness landscape is an operational concept used in evolutionary biology to visualize the relationship between the genotype constellation of an RVA strain and its fitness (ability to replicate and transmit) in a given environment. The vertical arrow at the left indicates the fitness level, increasing from bottom to top, while the color gradient of the spheres represents the transition of the viral population across the landscape. Peaks represent high fitness, while valleys represent low fitness. Created anew for this article.

To re-examine and address this issue, introducing the concept of the fitness landscape may be helpful. The interpretation of the CU-1/K9/A79-10/Cat97 strains as being adapted to a single, combined homologous host (dogs or cats) hinges on whether a rotavirus can achieve dual-species adaptation or successful low-level circulation after a host jump. When an RVA jumps from a cat (its natural homologous host) to a human (a heterologous host), the virus immediately faces a fitness valley—a region of low viability in the evolutionary landscape (Figure 2). The initial mutations required for the RVA to successfully replicate, spread, and evade the immune system in the new species often cause an immediate, sharp decrease in fitness, as these necessary genetic changes may temporarily impair essential viral functions optimized for the original host. For the RVA to become truly adapted to the new host, it must acquire a specific set of fit genes either by accumulation of point mutations or more frequently by genetic reassortment [54]. Critically, these subsequent beneficial mutations only become available after the initial detrimental changes are fixed, meaning that the virus must first cross the fitness valley of low reproductive success before ascending a new fitness peak corresponding to successful adaptation in the heterologous host species.

#### 5.2.2. Spillover Events in Humans

It is reasonable to suppose that HCR3A or Ro1845 represented single spillover events without subsequent human-to-human spread, as evidenced by the very low detection rates of this specific constellation of RVA strains in surveillance samples. However, based on the available whole-genome sequence data, it is difficult to ascertain whether, for example, Cat97 resulted from a spillover infection from a CU-1-like canine rotavirus or, conversely, CU-1 resulted from a spillover infection from a Cat97-like feline rotavirus.

#### 5.2.3. Sustained Interindividual Transmission Among Cats and Dogs

A notable characteristic of homologous strains is their ability for efficient individual-to-individual transmission, which is demonstrated by a small infectious dose and the achievement of high viral titres in the homologous host as evidenced in the mouse and rabbit rotavirus model [55,56,57]. From this point of view, the findings regarding FRV64 isolated in a stray cat in Kagoshima, Japan, in 1990 [58] are noteworthy. FRV64 possessed the same genotype constellation with Cat97 with high nucleotide sequence identities (95.9–98.3%) [28]. During the same season and in the same locale, additional strains including FRV72 and FRV73 were isolated from cats that were not from the same litter [58]. Upon whole-genome sequencing, the tree strains had >99% identical sequences across the 11 genome segments [16]. While limited to a specific time and geographic location, the detection of virtually identical genomes in epidemiologically unlinked cats suggests—at a minimum—local cat-to-cat transmission chains. When viewed alongside the discovery of the genetically similar Cat97 strain in a different timeframe, these data support the hypothesis that Cat97-like strains are indigenous to the feline species.

#### 5.2.4. Different Behaviours of P[3] and P[9] Strains in Dogs

Conversely, only RVA strains possessing G3P[3] have been detected in dogs thus far and their genotype constellations are identical or very similar to those of Cat97/K9/CU-1 (Table 3). Thus, it is most reasonable to assume that these Cat97/K9-like strains are indigenous to dogs (homologous strains). Therefore, one must assume that the fitness valley for Cat97/K9-like strains between feline and canine hosts is very shallow, enabling these strains to adapt to two biologically very different host species.

However, it should be noted that dogs are not the homologous host to FRV-1/AU-1-like G3P[9] strains as such strains have never been detected in dogs. Since the VP8* subunit of the VP4 spike protein is involved in the initial binding to the host cell receptor [59], the difference in P genotype is likely to determine host selection; this hypothesis is supported by the classification of P[9] and P[3] into different P genogroups, P genogroup [II] and P genogroup [I], respectively [60,61].

## 6. Distinguishing Between a Spillover Event and Sustained Human-to-Human Transmission

### 6.1. The Importance of Distinction

The fundamental challenge remains: it is critically important to distinguish between a spillover event (a dead-end infection) and sustained human-to-human transmission when the detection rate of feline-like strains in human children is consistently low (1–2%) [62,63], yet never truly rare. This distinction has profound implications for understanding RVA evolution and public health risk because, if interspecies transmission is limited to the individual patient, it lacks any long-term, inheritable consequences and does not impact evolution.

### 6.2. The Question Restated

Thus, the finding that the isolation of AU-1 from a sick child as a result of direct interspecies transmission immediately raised a pivotal question: did AU-1 represent a single, incidental spillover event from the feline host to a child, or did it indicate that feline-like rotavirus strains were already circulating at a low frequency within the human population? In terms of the fitness landscape concept, the question can be restated as which category the AU-1-like genotype constellation currently falls into:

- A “Dead-End Spillover” (low fitness): The strain was transmitted directly, but its fitness in humans is too low to sustain circulation. This supports the view that AU-1-like strains are just sporadic events.
- A “Shoulder” of a Peak (moderate fitness): The strain can cause infection but has limited ability to transmit beyond the initial host. This suggests only limited spread.
- A “New Peak” (high fitness): The strain has successfully adapted to humans and is capable of sustained human-to-human transmission, justifying its classification as a new human genotype constellation.

### 6.3. Evidence of Human-to-Human Transmission and Reassortment

#### 6.3.1. AU-1/FRV-1-like Strains in Brazil

Suggestive evidence that AU-1/FRV-1-like strains circulate among humans at least a few cycles beyond their initial spillover events from cats to humans comes from numerous cases in Brazil. In one surveillance study [64], P[9] rotaviruses (the feline-derived genotype) were detected in 16 (10.2%) of 157 samples from diarrhoeal children. While six of these were G3P[9], novel reassortant combinations, specifically G1P[9] and G9P[9], were also found. These novel combinations suggest reassortment events occurred after the original feline-derived AU-1/FRV-1-like strains jumped to humans. Tsugawa et al. [36] determined the whole-genome sequences of eight of these Brazilian P[9] strains. Four G3P[9] strains (R47, R55, R57, and R142) were found to possess the full AU-1/FRV-1 genotype constellation. The identification of these Brazilian strains was a significant finding, as AU-1 had been the only whole-genome sequence of its kind available. After this long absence, another human strain, WZ606, possessing the same genotype constellation was isolated in Zhejiang, China, in 2013 and was published in 2016 [37] (Table 3). The whole-genome information about the AU-1/FRV-1-like strains increased when full-FRV-1-like genotype constellations from three cats were added to the list (FRV317, FRV348 and FRV384) (Table 3) [16].

#### 6.3.2. AU-1/FRV-1-Based Reassortants in Brazil and Japan

The remaining four strains from Brazil were two G1P[9] strains (R70 and R49) and two G9P[9] (R138 and R135), and they were shown to be reassortants with locally circulating human RVA strains possessing the Wa-like or DS-1-like genotype constellation [36] (Table 3). These observations in Brazil are paralleled by the isolation of RVA strain K8 (G1P[9]) from a 14-year-old child in Hokkaido, Japan, in 1977 [65]. Strain K8 was later characterized as a reassortant between strains belonging to the AU-1 and Wa genogroups [38] (Table 3). Crucially, K8 was obtained from a community outbreak of gastroenteritis [65], which is highly suggestive that the strain was spreading from human to human.

The existence of reassortment is a powerful observation. It requires two genetically distinct viruses (the feline-derived P[9] strain and the indigenous human strain) to infect the same human cell simultaneously. All these findings cannot be understood without supposing the existence of human-to-human transmission of FRV-1/AU-1-like strains, thereby arguing against the hypothesis that a low detection rate of AU-1-like strains (1–2%) is explained solely by independent, dead-end spillover events.

#### 6.3.3. Low-Frequency Circulation of AU-1/FRV-1-like Strains in Humans

The consistent detection of strains with the AU-1-like genotype constellation and evidence of reassortment with co-circulating human RVA strains suggest the AU-1/FRV-1-like virus has successfully passed the lowest point of the fitness valley following the feline-to-human jump.

**Figure 3:**
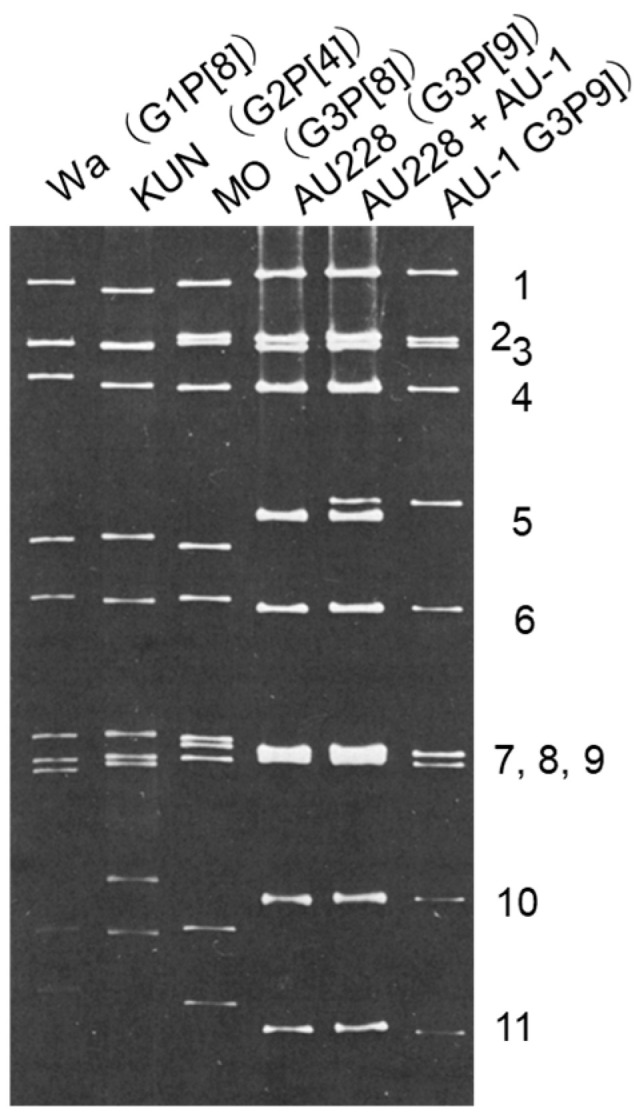
Polyacrylamide gel electrophoresis of genomic RNAs of AU-1-like strains in comparison with those of reference human RVA strains of the Wa-like and DS-1-like genotype constellation. RNA segments are indicated to the right of the gel. The characteristic RNA migration pattern of AU-1-like strains, as seen on the lane loaded with RNA from the AU-1 and AU228 strains, is defined by the well-separated migration of both the 5th and 6th genome segments and the 10th and 11th genome segments. Reproduced from ref. [66] with permission from the publisher.

Addressing the issue of FRV-1/AU-1-like strain circulation at a low level within the human population requires quantitative methods, yet this effort is immediately hindered by the difficulty of distinguishing these strains among the overwhelming abundance of indigenous human rotavirus strains. To circumvent this identification challenge, a simple, reproducible methodology involves utilizing a distinct electropherotype pattern, a technique often requiring expert visual interpretation. This tell-tale pattern is characterized by a long RNA migration profile with visibly separated 5th and 6th genome segments, in addition to wide separation between the 10th and 11th genome segments, as visualized upon polyacrylamide gel electrophoresis [64,66] (Figure 3).

#### 6.3.4. Examples of High Prevalence of P[9] Strains

Although the genotype constellation was unknown, it merits mention that far higher detection rates of P[9] strains were observed elsewhere in the world. For example, Mahmoud et al. [67] reported that RVA samples with P[9] were detected in 21 (24.7%) of 85 genotyped specimens collected in one children’s hospital in Egypt between January 2023 and March 2024, comprising G9P[9] and G4P[9] but no G3P[9]. Similarly, Piekarska et al. [68] reported that G3P[9] accounted for as many as 60% (41/68) of RVA-positive samples consecutively collected from children hospitalised in Lodz, Poland, between January and March 2010. These high detection rates of P[9] strains are highly suggestive of a feline RVA origin and underscore the certainty of human-to-human transmission.

## 7. Proposal to Define Phylogenetic Evidence for Interspecies Transmission

To definitively support the occurrence of a recent interspecies transmission event involving a particular RVA strain, sequence analysis must go beyond simple sharing of the identical genotype constellation. It is crucial to demonstrate not only that the sequences from the two strains (e.g., from feline and human hosts) share the same genotypes across all 11 genes but also that, in each gene, the two sequences belong to the same monophyletic lineage supported by a sufficiently high bootstrap probability (e.g., 80% or more) within a robust phylogenetic analysis. In parallel with the within-genotype diversity being approximately less than 20% [4], a within-lineage diversity of around less than 5% may well be appropriate as the subgenomic phylogeny, which was largely achieved in the subgenotype phylogeny for DS-1-like backbone genes [69].

Furthermore, assuming that the evolutionary rate of the genes in question is not significantly slower than generally accepted RVA evolutionary rates (e.g., 1 × 10^−3^ per site per year), high sequence identity (e.g., 95% or more) between two strains isolated from different animal species provides strong evidence that the interspecies transmission event occurred in the recent past. A minimum of 99% nucleotide sequence identity across all 11 genome segments may be required for inferring contemporary interspecies transmission, which corresponds to a most recent common ancestry within a few years. Supportive evidence for the most recent common ancestry may be obtained by constructing a maximum clade credibility tree using Bayesian Markov chain Monte Carlo analysis such as the one implemented in BEAST [70,71]. However, applying such stringent conditions requires the availability of relevant RVA strains from animal hosts, which are inherently underrepresented in surveillance focused on economic needs, not least because cats were shown to not to be associated with the presence of diarrhoea in a large epizootiologic study conducted in the United Kingdom [53].

## 8. Conclusions

The advent of metagenomics, whole-genome sequencing, and advanced phylogenetic analyses has raised the question of whether criteria not significantly advanced beyond the older operational model of genogrouping are still adequate. The documented cases of human RVA strains sharing entire genotype constellations with feline and canine strains (AU-1/FRV-1-like, Cat97-like) clearly demonstrate that interspecies transmission occurs. Furthermore, the observation of reassortment between feline-derived P[9] strains and indigenous human RVA strains argues against the idea that the circulation of these feline-like RVA strains is limited only to sporadic, dead-end spillover events. The existence of reassortment suggests the virus has successfully crossed the fitness valley, partially supporting the idea of onward human-to-human transmission following spillover events. However, definitive confirmation of this sustained transmission, or repeated, contemporary spillover events, may require adherence to more stringent phylogenetic criteria: the isolation of multiple human-derived strains possessing virtually identical sequences (e.g., >99%) within a monophyletic lineage supported by robust phylogenetic analysis. Conversely, the demonstration of definitive contemporary spillover requires identifying at least one pair of human and feline RVA strains that shows virtually identical sequences belonging to a monophyletic lineage across all genome segments. The status of the AU-1-like genotype constellation as a third, low-frequency human RVA genotype constellation awaits further studies that apply these new criteria to show such definitive evidence. The inherent underrepresentation of animal RVA strains in surveillance remains the primary challenge for robust comparison and confirmation.

## Figures and Tables

**Table 1:** Rotavirus proteins and their genotypes.

Viral Protein	Function	Genotype	Number of Genotypes Assigned	Cutoff Value (%)
VP7	**G**lyco-protein	Gx	42	80
VP4	**P**rotease-sensitive (=VP8* + VP5*)	Px	58	80
VP6	**I**nner-capsid	Ix	32	85
VP1	**R**NA-dependent RNA polymerase	Rx	24	83
VP2	**C**ore protein	Cx	24	84
VP3	**M**ethyl-transfer**ase**	Mx	24	81
NSP1	Interferon **A**ntagonist	Ax	39	79
NSP2	**N**TPase	Nx	28	85
NSP3	**T**ranslation enhancer	Tx	28	85
NSP4	**E**nterotoxin	Ex	32	85
NSP5/6	p**H**osphoprotein	Hx	28	91

The bold letter in each column of Function indicates the letter used to indicate the genotype. The x in the genotype column represents the number allocated for each specific genotype. The number of genotypes shown in the column is current as of this writing. An asterisk attached to VP5* and VP8* indicates that they are modified post-translationally.

**Table 2:** Two major and one minor genotype constellations of human RVAs and those for bovine and porcine RVA strains.

Prototype	VP7 (G)	VP4 (P)	VP6 (I)	VP1 (R)	VP2 (C)	VP3 (M)	NSP1 (A)	NSP2 (N)	NSP3 (T)	NSP4 (E)	NSP5 (H)
Wa-like genotype constellation	G1-4,9	P[8]	I1	R1	C1	M1	A1	N1	T1	E1	H1
DS-1-like genotype constellation	G2	P[4]	I2	R2	C2	M2	A2	N2	T2	E2	H2
AU-1-like genotype constellation	G3	P[9]	I3	R3	C3	M3	A3	N3	T3	E3	H3
Bovine genotype constellation	G6/8/10	P[1/5/11]	I2	R2	C2	M2	A3/13	N2	T6	E2	H3
Porcine genotype constellation	G3/4/5/9/11	P[6/7/19/23]	I1/5/12	R1	C1	M1	A1/8	N1	T1	E1	H1

## Data Availability

The present work constitutes a review and synthesis of the previously published literature. Consequently, all data supporting the figures and conclusions drawn in this article are available in the cited publications. No primary data were created during the research and writing of this review.

## Abbreviations

The following abbreviations are used in this manuscript:

RVA	Rotavirus A
VP	Viral protein
NSP	Nonstructural protein
